# Novel strategy for oral peptide delivery in incretin-based diabetes treatment

**DOI:** 10.1136/gutjnl-2019-319146

**Published:** 2019-08-10

**Authors:** Yining Xu, Matthias Van Hul, Francesco Suriano, Véronique Préat, Patrice D Cani, Ana Beloqui

**Affiliations:** 1 Advanced Drug Delivery and Biomaterials, Louvain Drug Research Institute (LDRI), UCLouvain, Université catholique de Louvain, Bruxelles, Belgium; 2 Metabolism and Nutrition Research group, Louvain Drug Research Institute (LDRI), UCLouvain, Université catholique de Louvain, Brussels, Belgium; 3 WELBIO, Walloon Excellence in Life Sciences and BIOtechnology, Brussels, Belgium

**Keywords:** nanocarriers, diabetes, GLP-1, steatosis, bioavailability

## Abstract

**Objective:**

To fulfil an unmet therapeutic need for treating type 2 diabetes by developing an innovative oral drug delivery nanosystem increasing the production of glucagon-like peptide-1 (GLP-1) and the absorption of peptides into the circulation.

**Design:**

We developed a nanocarrier for the oral delivery of peptides using lipid-based nanocapsules. We encapsulated the GLP-1 analogue exenatide within nanocapsules and investigated in vitro in human L-cells (NCl-H716) and murine L-cells (GLUTag cells) the ability of the nanosystem to trigger GLP-1 secretion. The therapeutic relevance of the nanosystem in vivo was tested in high-fat diet (HFD)-induced diabetic mice following acute (one administration) or chronic treatment (5 weeks) in obese and diabetic mice.

**Results:**

We demonstrated that this innovative nanosystem triggers GLP-1 secretion in both human and murine cells as well as in vivo in mice. This strategy increases the endogenous secretion of GLP-1 and the oral bioavailability of the GLP-1 analogue exenatide (4% bioavailability with our nanosystem).

The nanosystem synergizes its own biological effect with the encapsulated GLP-1 analogue leading to a marked improvement of glucose tolerance and insulin resistance (acute and chronic). The chronic treatment decreased diet-induced obesity, fat mass, hepatic steatosis, together with lower infiltration and recruitment of immune cell populations and inflammation.

**Conclusion:**

We developed a novel nanosystem compatible with human use that synergizes its own biological effect with the effects of increasing the bioavailability of a GLP-1 analogue. The effects of the formulation were comparable to the results observed for the marketed subcutaneous formulation. This nanocarrier-based strategy represents a novel promising approach for oral peptide delivery in incretin-based diabetes treatment.

**Significance of this study**

**What is already known on this subject?**

- Subcutaneously injected glucagon-like peptide-1 (GLP-1) analogues improve glucose metabolism and energy metabolism.
- Nanocarriers are promising tools to protect peptides from degradation or to increase their absorption across the grastrointestinal barrier.
- We recently discovered that specific lipid-based nanocapsules trigger endogenous GLP-1 secretion in vivo in mice when presenting a size of ∼220 nm.

**What are the new findings?**

- We developed a nanosystem which directly triggers endogenous GLP-1 secretion both in vitro on enteroendocrine L-cells (human and murine) and in vivo in obese and type 2 diabetic mice.
- We demonstrated that lipid nanocapsules remained stable and prevented exenatide degradation in vitro in biomimetic gastrointestinal fluids.
- We proved the proof-of-concept that oral delivery of nanocarriers can be used to increase the oral bioavailability of a GLP-1 analogue which must in theory be administered subcutaneously.
- This novel nanosystem acts synergistically by stimulating the endogenous production of GLP-1 secretion and allowing intestinal delivery of a GLP-1 analogue.
- The combination of the nanocarrier with a GLP-1 analogue has therapeutic effects on obesity, type 2 diabetes, dyslipidaemia and hepatic steatosis.
- We have conceptually chosen scalable preparation procedures and *generally recognised as safe* excipients that could be directly transferred to the pharmaceutical market.

**Significance of this study**

**How might it impact on clinical practice in the foreseeable future?**

- This novel nanosystem can be used for the development of oral delivery of different peptidic drugs (including GLP-1/GLP-2 analogues, or others) that are in theory injected.
- The cost-effectiveness as well as the compliance for the administration of drugs can be improved by using this innovative nanosystem.
- The capacity of this novel nanocarrier to stimulate the endogenous secretion of glucagon-like peptides can be exploited in different pathological situations such as obesity/diabetes but also inflammatory bowel diseases (eg, GLP-2 secretion).

## Introduction

The development of oral dosage forms that enable the absorption of therapeutic peptides into the systemic circulation is one of the greatest challenges for the pharmaceutical industry.^1^ Selected diabetes peptides have transitioned to the late phase of development despite their low oral bioavailability (estimated to be between 0.5% and 1.0%).^2^ Nevertheless, the benefits of oral peptide delivery over an intravenous or subcutaneous administration are remarkable, and especially in the case of anti-diabetic drugs, for example, glucagon-like peptide-1 (GLP-1).^3^ The oral administration of incretin mimetic peptides has the additional therapeutic advantage of simulating the normal physiological pathway of the native peptide.^4^ GLP-1 agonists target the liver, which can be accessed in much higher concentrations via the hepatic portal vein than via subcutaneous delivery, thereby reducing systemic exposure and its associated side effects.^5^ Despite the numerous ongoing efforts to exploit the oral route of administration of incretin mimetic peptides, only two GLP-1 analogues are currently administered orally in clinical trials,^1 3^ and both of these require coadministration with functional excipients.^3^

The gut physiology offers a stimulating environment, which has not yet been fully exploited to its full potential in the drug delivery field. Enteroendocrine L-cells have attracted particular interest because of the pleiotropic effects of their secreted peptides (eg, GLP-1 and GLP-2).^6^ These cells are attractive targets for the treatment of diseases such as type 2 diabetes (T2DM) and inflammatory bowel diseases (IBD).^7^ In the context of T2DM, GLP-1 secreted from intestinal L-cells stimulates postprandial insulin secretion and is quickly hydrolysed by dipeptidyl peptidase-IV (DPP-IV).^8^ Thus, several GLP-1 analogues with improved plasma half-life (eg, exenatide, semaglutide) have been developed and proven to be successful for treating T2DM. Recently, researchers have turned their attention from the secreted peptides towards the L-cells themselves. Indeed, enhancing endogenous GLP-1 secretion would represent a novel alternative in incretin-based diabetes therapy.^9–11^ We hypothesised that using nanocarriers targeting intestinal L-cells could represent an alternative therapeutic strategy to stimulate the production of gut peptides. Nanocarriers could be engineered to simulate certain ligands and can be designed to have increased gastrointestinal retention, thereby evoking long-term activation of L-cells.^6^ In this context, a major breakthrough of our research was proving the ability of lipid-based nanocapsules (LNC) to trigger endogenous GLP-1 secretion in vivo after oral administration in mice.^12–14^ However, despite these encouraging results and the promising properties attributed to nanomedicines, we need to prove that these novel formulations perform better than standard formulations incorporating functional excipients in oral peptide delivery.

Here, we report an innovative nanocapsule-based drug delivery system that synergizes its own biological effect (stimulation of GLP-1 release) and that of the encapsulated bioactive molecule (exenatide) as an alternative strategy for the treatment of T2DM and hepatic steatosis via an oral route.

## Results

### Exenatide is successfully encapsulated and preserved within LNC

We selected LNC as nanocarriers for the oral delivery of peptides. We recently discovered that these nanocapsules triggered endogenous GLP-1 secretion in vivo in mice when presenting a size of ∼200 nm.^14^ Therefore, we hypothesised that synergizing the biological effect of LNC together with the pharmacological effects of an encapsulated GLP-1 analogue could be highly innovative. As a proof of concept, we selected the GLP-1 analogue exenatide (EXE) as the incretin mimetic to be encapsulated within LNC containing reverse micelles (RM-LNC). The final composition of the formulation is described in online supplementary table S1, and the physico-chemical characterisation is detailed in online supplementary table S2. A schematic representation of EXE-RM-LNC is depicted in online supplementary figure S1. Notably, EXE-RM-LNC exhibited an entrapment efficiency of ~85%. It is worth noting that we already considered the potential translational approach; therefore, we have conceptually chosen scalable preparation procedures and *generally recognised as safe* (GRAS) excipients that could be directly transferred to the pharmaceutical market.

10.1136/gutjnl-2019-319146.supp1 Supplementary data

Lipid nanocapsules remained stable and prevented exenatide degradation in vitro in biomimetic gastrointestinal fluids (online supplementary figure S2). The stability of the nanocapsules was confirmed in five biomimetic media, including fasted-state and fed-state simulated gastric or intestinal fluids (FaSSGF with or without pepsin, FaSSIF, FeSSIF and FeSSIF-v2, respectively) (online supplementary figure S2a-e). A detailed description of the composition of the biomimetic media used is described in online supplementary table S3.

These results confirmed that the newly developed nanocapsules retained the same gastro-resistant properties as previously described conventional LNC.^14 15^ The in vitro exenatide release profile was further evaluated in gastric medium (FaSSGF without pepsin, pH 1.6) and intestinal medium (FaSSIF, pH 6.5). Exenatide was progressively released from the RM-LNC over a 6-hour period, reaching a cumulative exenatide release of 60% after this time in FaSSIF, which was undetectable in FaSSGF (online supplementary figure S2f).

### RM-LNC induce GLP-1 secretion both in vitro and in vivo and increase exenatide blood levels in normoglycaemic mice

**Figure 1:**
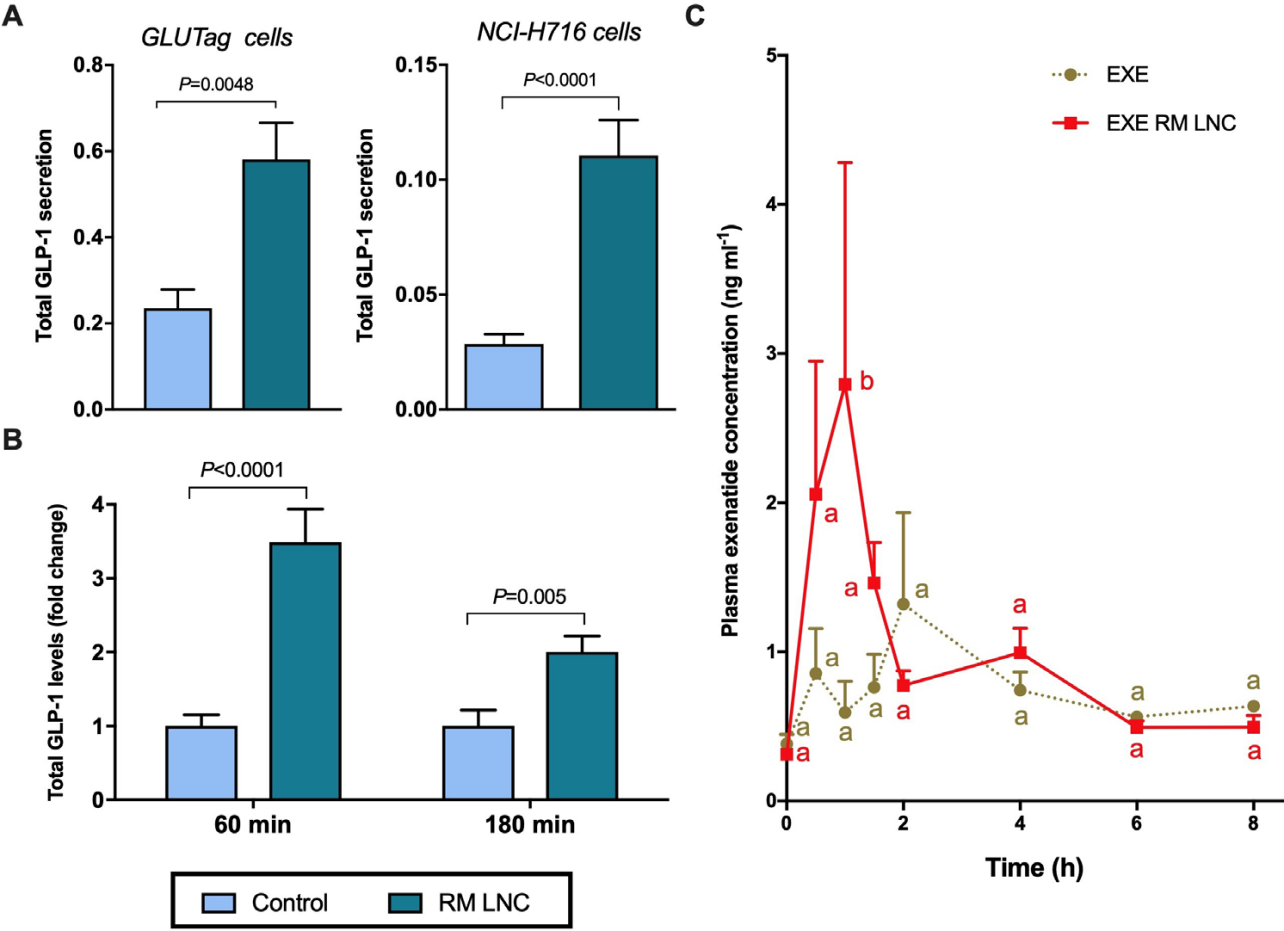
RM-LNC-mediated glucagon-like peptide-1 (GLP-1) secretion in vitro and in vivo and exenatide plasma levels in normoglycaemic mice. (A) RM-LNC-mediated in vitro GLP-1 secretion (2 mg/mL) in GLUTag (left) and NCI-H716 (right) cells (murine and human L-cells, respectively) after a 2-hour coincubation period (mean±SEM; n=6–10). (B) In vivo total GLP-1 secretion in normoglycaemic mice 60 and 180 min after RM-LNC oral administration (mean±SEM; n=7–8). (C) Exenatide blood profile after oral administration to normoglycaemic mice in solution in water (EXE) or encapsulation within RM-LNC (EXE-RM-LNC) (500 µg/kg exenatide dose) (mean±SEM; n=4). P values in (A, B) were determined by Student’s t test or Mann-Whitney test. Data with different superscript letters (C) are significantly different (p<0.05) according to two-way analysis of variance followed by Tukey’s post hoc test.

First, we investigated the ability of RM-LNC on their ability to trigger GLP-1 secretion in vitro in both murine L-cells (GLUTag cells) and in human L-cells (NCl-H716) (figure 1A). We found that RM-LNC of particle size ∼220 nm were able to trigger endogenous GLP-1 secretion in both in vitro models (figure 1A).

Second, we investigated whether RM-LNC could trigger GLP-1 secretion in vivo in normoglycaemic mice (figure 1B). Notably, the pharmacological effect was preserved in vivo because following the oral administration of RM-LNC, we found that the GLP-1 levels were increased up to ∼ threefold. Therefore, the pharmacological effect of the nanocarriers was reproduced in vitro in both cell lines irrespective of the nature of the cells (murine or human) and in vivo in normoglycaemic mice.

To validate our hypothesis that RM-LNC not only trigger the endogenous secretion of GLP-1 but also act as a dual-action nanocarrier for the oral delivery of incretin mimetic peptides, we needed to prove the ability of the nanocarriers to increase their absorption. For this purpose, we measured exenatide plasma concentration on oral administration as a solution in water or encapsulated within RM-LNC (500 µg/kg exenatide dose) (figure 1C). Exenatide plasma levels were higher when encapsulated within RM-LNC than when delivered as a solution. Altogether, these data confirmed our hypothesis regarding the efficacy of RM-LNC at both enhancing endogenous GLP-1 levels and increasing EXE plasmatic levels. Complementary data on GLP-1 extracellular and intracellular levels measured in murine and human L-cells in vitro are supplied in online supplementary figure S3. Of note, we found no evidence of cytotoxicity in human intestinal Caco-2 cells or NCI-H716 cells (online supplementary figure S4a-d).

### Combination of endogenous GLP-1 release with increased exenatide plasma levels improves glycaemia in diabetic mice

**Figure 2:**
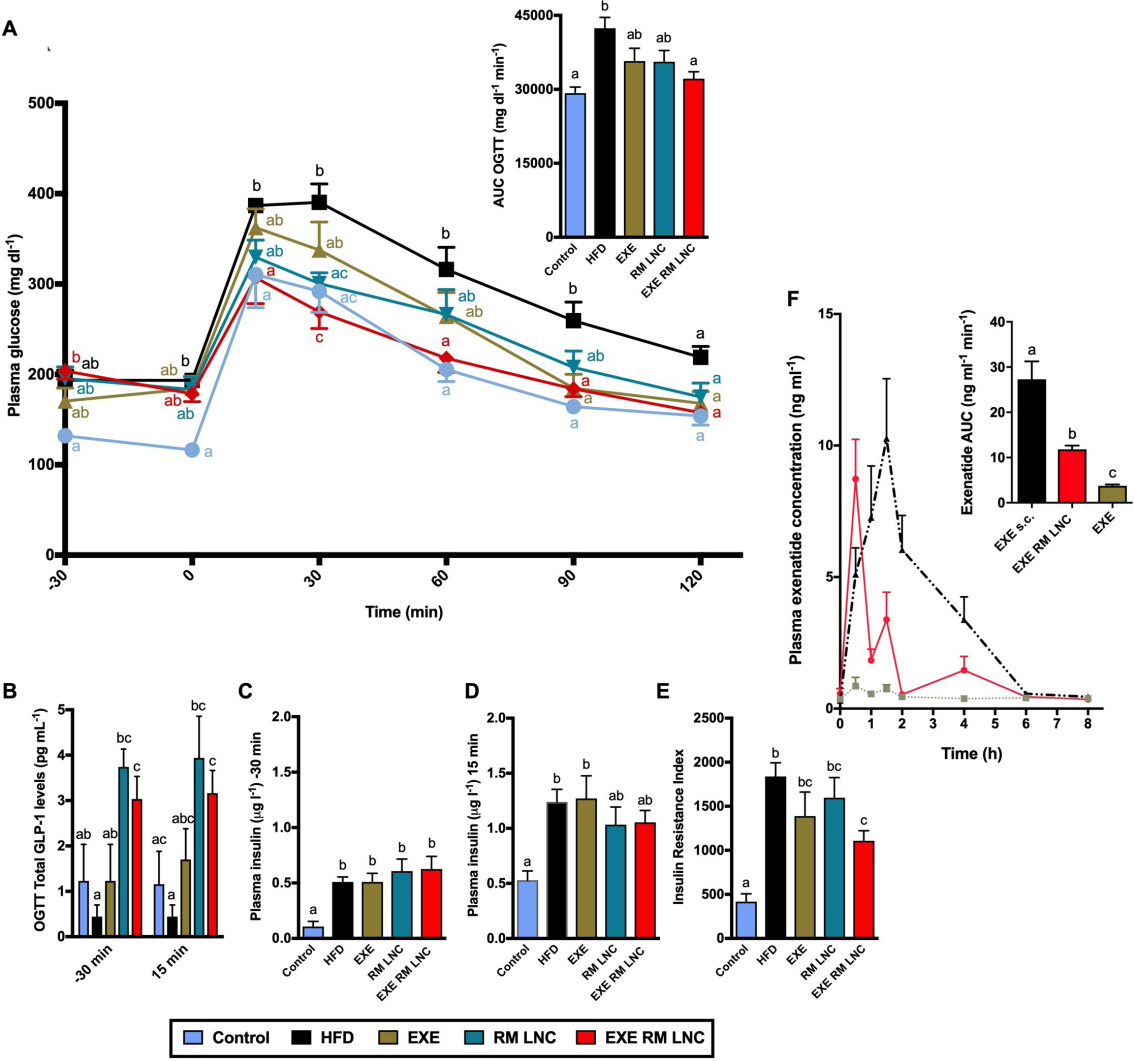
Pharmacodynamic and pharmacokinetic evaluation of EXE-RM-LNC in a high-fat diet (HFD)-induced obese/diabetic mouse model following acute treatment. (A) Plasma glucose levels (mg dL^-1^) measured 30 min before and 120 after glucose challenge (n=8–9) and mean area under the curve (AUC, mg dL^-1^ min^-1^) measured 30 min before and 120 after glucose challenge (n=8–9). (B) Plasma total glucagon-like peptide-1 (GLP-1) levels and (C, D) plasma insulin levels measured 30 min before and 15 min after glucose challenge (n=8–10). (E) Insulin resistance index (n=8–9). (F) Concentration-time profile and AUC of exenatide after subcutaneous administration (EXE s.c.) (50 µg/kg exenatide dose) and oral administration, in solution and within RM-LNC (EXE and EXE-RM-LNC, respectively) (500 µg/kg exenatide dose). Data are presented as the mean±SEM (n=8–10). Data with different superscript letters (A, B) are significantly different (p<0.05) according to a two-way analysis of variance (ANOVA) followed by Tukey’s post hoc test or (C–F) according to a one-way ANOVA followed by Tukey’s post hoc test.

The therapeutic relevance regarding the combination of nanocarrier-mediated endogenous GLP-1 levels with increased exenatide plasma concentration was evaluated in a diet-induced obese/diabetic mouse model. As a proof-of-concept study, we tested EXE-RM-LNC in a murine high-fat diet (HFD)-induced T2DM model (figure 2) following acute treatment (one single administration). First, we confirmed that the HFD mice were markedly hyperglycaemic and hyperinsulinemic in fasted state (figure 2A, C, D) and displayed a strong degree of insulin resistance (eg, index of insulin resistance) (figure 2E).

We administered once orally a 500 µg/kg exenatide dose (free and encapsulated within RM-LNC) and equivalent concentrations of RM-LNC or water 60 min prior to an oral glucose administration (2 g/kg) (corresponding to the time point of −60 min in figure 2A), this time point is based on the increased secreted GLP-1 levels measured in normoglycaemic mice (figure 1B).

Strikingly, we found that EXE-RM-LNC treatment completely normalised the glycaemia, as glycaemia in these mice followed the same profile throughout the overall oral glucose challenge as that observed in lean normoglycaemic control mice (figure 2A).

Conversely, the glucose levels measured in the EXE-treated mice remained similar to those in the HFD-fed mice at 30 min and then remained higher than those in the EXE-RM-LNC-treated mice until 90 min (figure 2A). EXE-RM-LNC were able to significantly decrease plasma glucose levels and glucose area under the curve (AUC) (figure 2A). Importantly, we found that total GLP-1 levels were significantly increased in both RM-LNC and EXE-RM-LNC-treated groups compared with the control groups (Control and HFD), confirming the ability of the nanosystem per se to stimulate GLP-1 release under pathological conditions (figure 2B).

Importantly, only EXE-RM-LNC significantly reduced the insulin resistance index compared with the HFD group (figure 2E).

Interestingly, RM-LNC alone had an effect on lowering blood glucose levels compared with untreated HFD mice. However, as we anticipated, this effect was not sufficient as per reducing the hyperglycaemia. A pharmacokinetic study measuring exenatide levels in HFD mice confirmed that exenatide blood levels were significantly increased when orally administered within RM-LNC (EXE-RM-LNC) compared with exenatide in solution (relative bioavailability 4.32% with LNC, p<0.001). This relative bioavailability can be considered a high value and a great improvement, considering that peptides presenting between a 0.5% and 1% bioavailability have progressed to the late phase of development.^2^ The calculated pharmacokinetic parameters are summarised in online supplementary table 4. We observed a different exenatide pharmacokinetic profile (eg, different C_max_, AUC, T_max_) in normoglycaemic (figure 1C) versus HFD mice (figure 2F).

Taking together the increased exenatide blood levels and the increased endogenous GLP-1 levels, these data serve as a strong proof of concept regarding the effectiveness of our nanosystem in ameliorating T2DM symptoms.

Based on the present data, we decided to further expand our findings by investigating the impact of EXE versus EXE-RM-LNC in mice treated for 8 weeks and 10 weeks with HFD. This model is a stronger model of diet-induced diabetes and metabolic disorders in mice. It is important to highlight that we obtained equivalent results regarding the efficacy of EXE-RM-LNC on the reduction of blood glucose levels during OGTT, regardless of the chronicity of the disease (online supplementary figure S5).

### Chronic EXE-RM-LNC long-term treatment improves glucose metabolism in obese and diabetic mice

To evaluate the impact of chronic and long-term treatment with EXE-RM-LNC on glucose metabolism, we induced obesity and type 2 diabetes in mice by treating them for 8 weeks with a HFD, then we continued the HFD for 5 weeks and treated the mice with daily administration of 500 µg/kg exenatide (oral) (EXE-RM-LNC) or equivalent amounts of unloaded nanocapsules (RM-LNC) or free exenatide (EXE) or water. To evaluated the impact of our experimental treatment with existing treatment strategies including a group treated with a marketed subcutaneous form of exenatide, Byetta. The oral administration of exenatide was compared with the subcutaneous injection (s.c.) of 10 µg/kg exenatide in solution or as Byetta.

**Figure 3:**
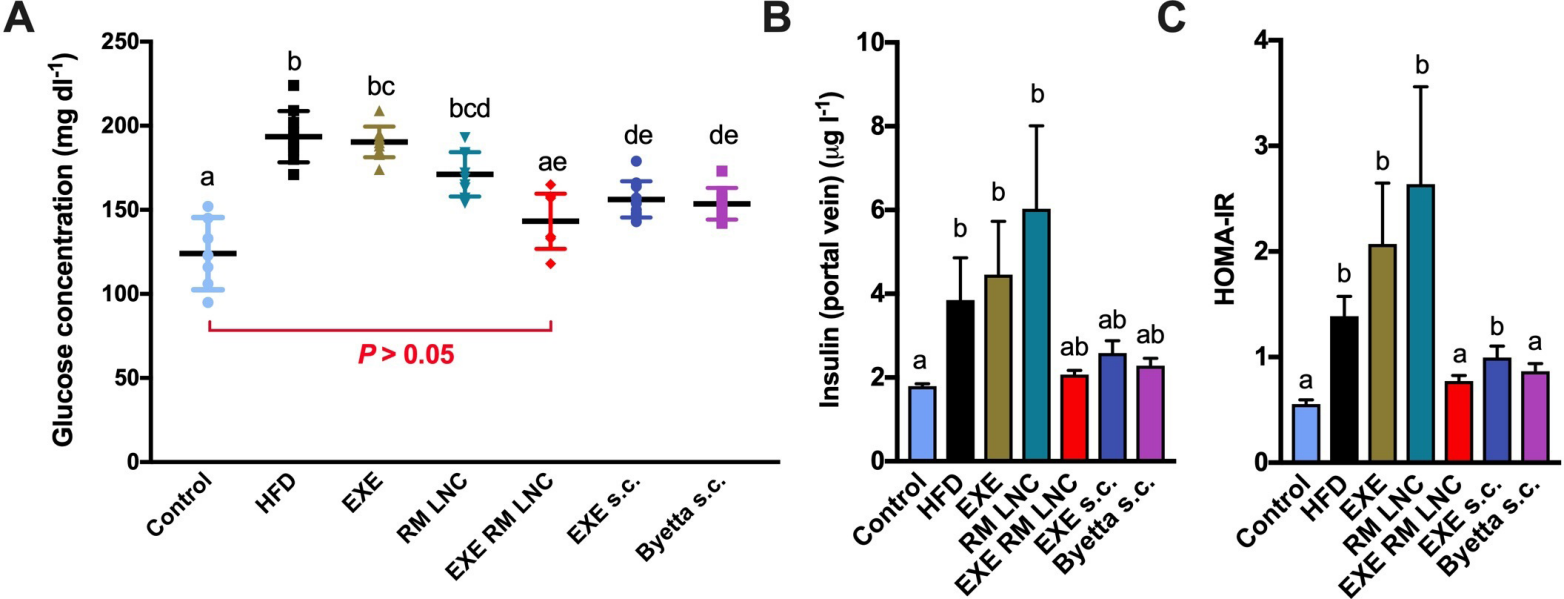
Effect of EXE-RM-LNC on glucose homeostasis and hyperinsulinemia in obese/diabetic mice. (A) Plasma glucose levels (mg dL^-1^) after 5 weeks of treatment (13 weeks of high-fat diet (HFD) feeding). Data are presented as the mean±SEM (n=10). (B) Insulin plasma levels measured from the portal vein (n=8–10). Data are presented as the mean±SEM data with different superscript letters are significantly different (p<0.05). (C) HOMA-IR was calculated using the equation [fasting glucose (mg/dL) × fasting insulin (ng/mL)]/405 as previously described.^27^ Data are presented as the mean±SEM (n=8–10). P values in (A) were determined by a two-way analysis of variance followed by Tukey’s post hoc test. P values in (B, C) were determined by Kruskal-Wallis followed by Dunn’s post hoc test.

After 5 weeks of daily treatment, mice were sacrificed, and blood was drawn from both the portal and the cava vein. Plasma glucose and insulin levels are depicted in figure 3A, B respectively. Interestingly, after 5 weeks of treatment, only the EXE-RM-LNC were able to decrease plasma glucose levels to reach levels comparable to that of the control group (figure 3A). It is noteworthy that EXE-RM LNC-treated mice had significantly lower plasma glucose levels than the RM-LNC-treated mice. Plasma glucose levels were also significantly lower in the RM-LNC group than in the EXE-treated. Therefore, these data demonstrate the synergistic effect provided by EXE-RM-LNC on glucose homeostasis. This effect is likely due to the combination of the endogenous GLP-1 secretion triggered by RM-LNC and elevated exenatide plasma levels. Additionally, EXE-RM-LNC-treated and subcutaneously treated mice exhibited comparable insulin levels as the control group. Although this reduction compared with the HFD group did not reach statistical significance when the data were analysed by the Kruskal-Wallis followed by Dunn’s post hoc test (figure 3B), a significant difference was found when analysed by the Mann-Whitney test (p=0.04 for EXE-RM-LNC vs HFD). HFD induce a massive insulin resistance (measured by using the homeostasis model assessment of insulin resistance (HOMA-IR)) which was completely normalised with the EXE-RM-LNC, an effect which was therapeutically equivalent to the marketed drug (Byetta) based on GLP-1 (figure 3C).

### Chronic treatment with EXE-RM-LNC decreases diet-induced steatosis in obese/diabetic mice

**Figure 4:**
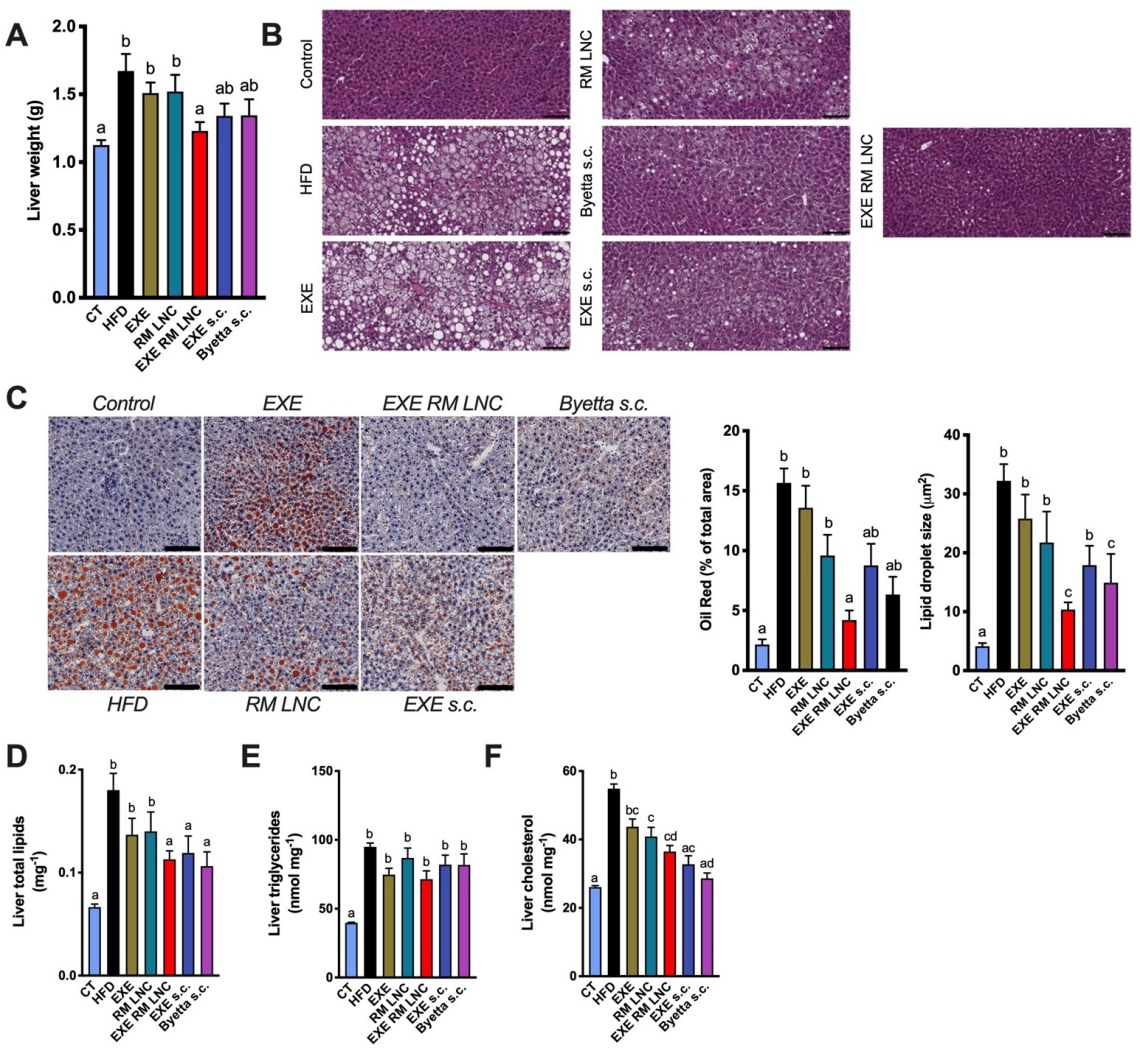
EXE-RM-LNC treatment impacts lipid homeostasis. (A) Liver weight (g). (B) Morphology of the liver in H&E-stained sections following a chronic/long-term EXE treatment in diabetic and obese mice. Representative liver H&E staining (scale bar: 100 µm). (C) Representative liver oil red O staining (scale bar: 100 µm), oil red O staining expressed in percentage of area stained and mean lipid droplet size (μm^2^, n=5–7). (D) Liver total lipid content (mg^-1^ per 100 mg of tissue). (E) Liver triglycerides (nmol mg^−1^). (G) Liver cholesterol (nmol mg^−1^). Data with different superscript letters (A–F) are significantly different (p<0.05). P values in (A) were determined following one-way analysis of variance with Tukey’s post hoc test. P values in (C–F) were determined by the Kruskal-Wallis test followed by Dunn’s post hoc test.

We found that the liver weight was only significantly lower in the EXE-RM-LNC-treated group after 5 weeks of treatment (13 weeks of HFD feeding) compared with the HFD-fed group (figure 4A). Histological analysis by H&E and oil red O staining revealed a marked decrease in hepatic steatosis, as evidenced by lower hepatic lipid accumulation and by fewer and smaller lipid droplets in EXE-RM-LNC-treated mice than in HFD mice (figure 4B, C). Despite the minor impact on triglyceride levels, hepatic total lipid content and cholesterol levels were comparable between EXE-RM-LNC-treated and subcutaneously treated mice and were significantly lower than those in the HFD group (figure 4D–F).

It is of the utmost importance to note that our innovative approach using EXE-RM-LNC was more efficient in decreasing liver weight than all the other treatments and was efficient as the marketed drug by the typical administration route (s.c.) (Byetta), thereby clearly showing the better effects on glucose parameters and a non-inferiority on liver markers compared with the subcutaneous approach.

**Figure 5:**
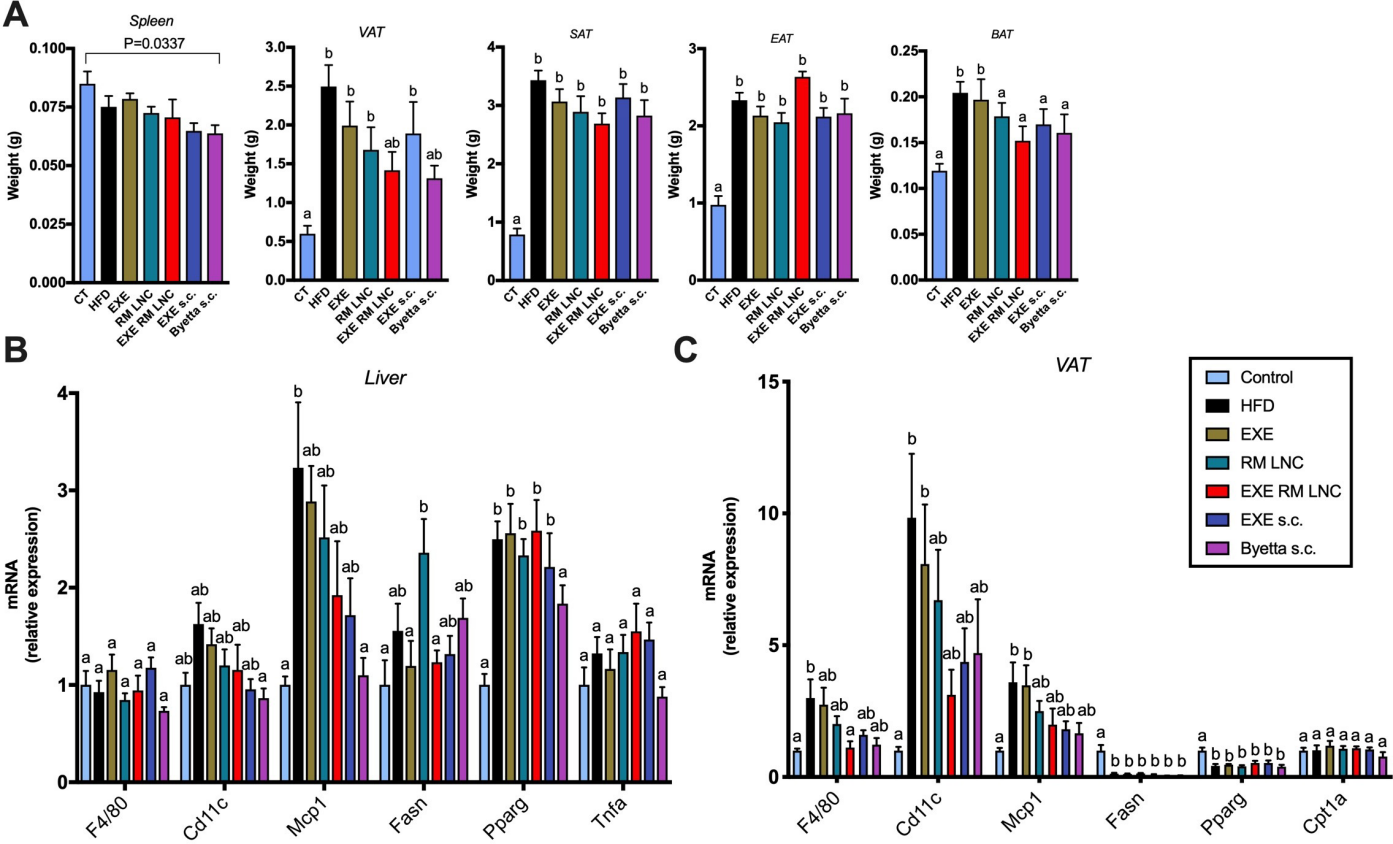
EXE-RM-LNC treatment impacts infiltration/recruitment of immune cell populations in the visceral adipose tissue. (A) Weights of the spleen, different white adipose tissues and brown adipose tissue (BAT) (g). Data are presented as the mean±SEM (n=9–10). (B) mRNA expression of *F4/80, Cd11c*, *Mcp1*, *Tnf*a, *Fasn* and *Pparg* in the liver (n=9–10). (C) mRNA expression of *F4/80, Cd11c*, *Mcp1*, *Fasn*, *Pparg* and *Cpt1*A in visceral adipose tissue (n=9–10). Data with different superscript letters (A–C) are significantly different (p<0.05). P values in (A, C) were determined following one-way analysis of variance (ANOVA) with Tukey’s post hoc test. P values in (B) were determined by a one-way ANOVA with Tukey’s post hoc test or the Kruskal-Wallis test followed by Dunn’s post hoc test. EAT, epididymal adipose tissue; SAT, subcutaneous adipose tissue; VAT, visceral adipose tissue.

In addition to the biochemical and histological analysis, key markers associated with infiltration/recruitment of immune cell populations (*F4/80, Cd11c, Mcp1*), inflammation (*Tnfa*) and lipid metabolism (*Fasn, Pparg, Cpt1a*) were analysed by quantitative PCR both in the liver and in visceral adipose tissue (VAT). Although we encountered no significant differences in the mRNA expression of *F4/80, Cd11c, Mcp1, Tnfa, Fasn* and *Pparg* in the liver by analysis of variance (ANOVA) (figure 5B) (large number of groups being compared), we found significant differences by the Mann-Whitney test. In the liver, *Cd11c* and *Mcp1* mRNA expression in EXE-RM-LNC-treated mice was significantly lower than that in the HFD group (P=0.05 and P=0.0355, respectively), and the same effect was observed in the Byetta-treated group (P=0.0015 and P=0.0007, respectively).

Visceral fat mass is considered a risk factor for developing liver diseases and insulin resistance.^16 17^ Hence, we found that both the EXE-RM-LNC- and Byetta-treated groups exhibited less fat than in HFD-fed mice, and there was not significant difference in the amount of fat in these groups from that in the untreated control group (figure 5A). Although their weights were not significantly different from the HFD group according to the Kruskal-Wallis analysis (large number of groups being compared), they were highly significantly different when analysed by the Mann-Whitney test (P=0.0057 for EXE-RM-LNC vs HFD and P=0.0004 for Byetta vs HFD). Interestingly, higher expression of *F4/80* observed in the HFD group was significantly downregulated in the visceral fat mass only in EXE-RM-LNC-treated mice following ANOVA. No significant effects were observed for the other markers when compared with the HFD group (figure 5C).

## Discussion

Despite numerous ongoing efforts, the transformation of injectable therapies for T2DM into oral drug delivery strategies remains a challenge. As a result, current treatments with GLP-1 analogues on the market are still administered exclusively subcutaneously. Current state-of-the-art strategies for oral peptide delivery use the delivery system merely as a vehicle, and none of them have explored the possibility that the carrier could have additional therapeutic effects on the final formulation. In the context of incretin-based diabetes disease therapy, the enhancement of endogenous GLP-1 secretion represents a novel alternative treatment that more closely resembles the physiology of the peptide.^18 19^ Here, we demonstrated the therapeutic effect of secreted endogenous peptides with encapsulated synthetic analogues within a lipid-based drug delivery system as an innovative approach for the oral delivery of peptides. Translational considerations have been conceptually integrated by choosing scalable preparation procedures and GRAS excipients that could be directly transferred into the pharmaceutical market.

We confirmed in vitro, both in murine and human L-cells, and in vivo in normoglycaemic mice, the ability of exenatide-loaded lipid nanocapsules to induce GLP-1 secretion. Additionally, we conducted a pharmacokinetic study confirming the absorption of exenatide into systemic circulation. After proving the stability of the formulation, the ability of the nanosystem to preserve peptide integrity, and its ability to increase GLP-1 levels in vivo while enabling the absorption of the peptide into systemic circulation, we demonstrated the efficacy of the formulation in the pathological context in an HFD-induced obese/diabetic mouse model.

The dual-action of the nanosystem for improving glycaemia was observed in vivo in obese/diabetic mice following acute or chronic treatment. A single acute administration of the nanosystem normalised blood glucose levels, which were comparable to those of the control group. Although both empty and EXE-loaded nanocapsules presented increased GLP-1 levels compared with untreated groups, only EXE-RM-LNC-treated mice exhibited a significant decrease in the insulin resistance index. Therefore, suggesting an additional mechanism of action. A pharmacokinetic analysis in obese/diabetic mice confirmed that EXE-RM-LNC increased exenatide bioavailability to more than 4%. These data served as a strong proof-of-concept regarding the effectiveness of a dual-action drug delivery nanosystem in ameliorating glycaemia by combining both increased endogenous GLP-1 levels and increased peptide bioavailability.

To demonstrate that this nanosystem represents a plausible alternative to current strategies for the oral delivery of incretin peptides in the treatment of T2DM, we conducted a chronic/long-term treatment consisting of a 5-week daily administration protocol. After 5 weeks of treatment, EXE-RM-LNC-treated mice exhibited normalised plasma glucose levels comparable to those of untreated control mice, along with decreased insulin levels. Therefore, the beneficial effects observed are not limited to the acute effect of the nanocarriers but are also translated to a therapeutic effect on oral glucose tolerance.

It is worth noting that our approach led to comparable results regarding glucose homeostasis to those observed for the current marketed drug that is administered subcutaneously. Therefore, our results demonstrate the non-inferiority of our approach together with the benefit of administration by the oral route for chronic treatments.

However, unlike subcutaneously treated mice, the metabolic effect observed for EXE-RM-LNC was decoupled from any effect on the total body weight (online supplementary figure 6). As both EXE-RM-LNC and the subcutaneously administered EXE decreased the visceral adipose fat mass and, to some extent, the liver lipid content, we cannot rule out the possibility that the metabolic effects were due to an impact on these organs (eg, visceral fat and liver lipid) rather than an overall change in body weight.

Our data point to a strong trend towards lower key markers associated with infiltration-recruitment of immune cell populations (macrophages, dendritic cells) and inflammatory markers in both the liver and VAT, again highlighting the beneficial effects of our approach on such markers. *F4/80* is a marker of inflammatory cell infiltration (mature macrophages), whereas *Cd11c*, *Mcp1* and *Tnfa* are known to reflect the M1 macrophage phenotype during obesity-associated inflammation. It is important to note that EXE-RM-LNC was the only treatment that significantly reduced macrophage infiltration in the VAT, a hallmark of chronic inflammation also considered as triggering factor for insulin resistance and diabetes.^20–22^ From a mechanistic point of view, we previously showed that RM-LNC increase the secretion of GLP-1 and, likely, the copeptide GLP-2, which has been shown to reinforce the gut barrier function, thereby reducing bacterial compound translocation, inflammation and steatosis in obese rodents.^23^ Whether the lower inflammation observed here could depend on a similar mechanism is plausible but remains to be demonstrated.

The mechanisms by which GLP-1 levels are increased via lipid nanocapsules remain unclear; however, we may not rule out that the daily oral gavage stimulates the production of the gut peptides throughout the day and, therefore, contributes to maintaining a better metabolic profile in the HFD-fed mice. Indeed, a daily chronic administration was sufficient to improve metabolism and even normalise other markers. Finally, we cannot exclude the possibility that lipid nanocapsules could modulate the activity of DPP-IV, thus preserving increased circulating total GLP-1 levels in the body, which warrants further investigation. Another important matter that will need further investigation is the potential toxicological impact of the formulation in the intestine on the accumulation of the formulation following repeated doses. It would be interesting to compare the potential toxicological effect of our formulation (providing with GLP-1 levels cleaved by DPP-IV enzyme, and reduced GLP-1 analogue doses) with the potential toxicological effect of the synthetic peptide administered alone, which has been modified not to be recognised by DPP-IV and therefore, could be potentially accumulated in the body.

In conclusion, we developed an innovative approach using incretin mimetics via the oral route. We discovered that combining nanocarriers with GLP-1 analogues is sufficient to normalise the glycaemia of obese/diabetic mice after either acute or chronic treatment. Interestingly, in addition to the strong advantage of using the oral route, this approach is at least as efficient as the current marketed drug as a comparison and could even be more potent for improving oral glucose tolerance, insulin resistance and hepatic steatosis. Thus, our strategy offers an additional advantage over current approaches for oral incretin mimetic peptide delivery and by increasing endogenous GLP-1 levels. All these developments could lead to enhanced clinical translation of nanomedicines in oral incretin-based T2DM treatment.

## Materials and methods

### Materials

#### Preparation of reverse micelle-loaded lipid nanocapsules

Reverse micelle-loaded lipid nanocapsules (RM-LNC) were formulated in two steps, in which the drug was first encapsulated within reverse micelles and then further encapsulated within LNC. First, exenatide-loaded reverse micelles (EXE-RM) were prepared by high-speed stirring of a surfactant (Span 80) and an oil (Labrafac WL 1349) mixture (1:5 wt ratio). Then, 50 µL of EXE (30 mg/mL in MilliQ water) was dripped into the mixture and maintained under stirring. Exenatide-loaded reverse micelle lipid nanocapsules (EXE-RM-LNC) were prepared following a modified phase inversion process described by Heurtault *et al*.^24^ Briefly, all components (shown in online supplementary table S1, figure 1C), including lipophilic Labrafac WL 1349, Peceol, Lipoid S100, Solutol HS15, sodium chloride (NaCl) and MilliQ water, were mixed together under magnetic stirring at 40°C at 200 rpm for 5 min. Temperature cycles of progressive heating/cooling were conducted between 50°C and 67°C. During the last cycle, 500 µL of preheated drug loaded RM was added to the mixture at ~3°C above the phase inversion zone (PIZ; 59°C–61.5°C). The solution was cooled to reach the PIZ temperature, and 2.5 mL of cold water (4°C) was added and stirred at high speed for 2 min. Blank RM-LNC were prepared following the same protocol in the absence of exenatide.

#### In vitro GLP-1 secretion

The in vitro GLP-1 secretion was quantified in both GLUTag (a kind gift from Professor Daniel Drucker, Toronto, Canada) and NCI-H716 cells as previously described by Xu *et al* 14.

#### Animals

All animal experiments were approved by and performed in accordance with the local animal committee (2014/UCL/MD/033 and 2017/UCL/MD/005) and as specified by the Belgian Law of 29 May 2013 on the protection of laboratory animals.

#### Oral glucose tolerance test in HFD induced obese/diabetic mice

Eight-week-old male mice were housed five per cage and divided into five groups (10 mice per group). After 2 weeks of acclimation, mice were fed a HFD (60% fat and 20% carbohydrates (kcal per 100 g), D12492i, Research Diets, USA) (HFD and exenatide-treated groups) or a normal control diet (control, AIN93Mi, Research Diets, USA) for 3, 8 or 10 weeks before experiments and underwent a period of fasting overnight before being treated with an oral exenatide solution (EXE, 500 µg of exenatide/kg body weight), exenatide-reverse micelle-loaded lipid nanocapsules (EXE-RM-LNC, 500 µg of exenatide/kg body weight) or unloaded micelle-loaded lipid nanocapsules (RM-LNC, equivalent concentrations as per EXE-RM-LNC) 1 hour before being challenged with an oral glucose gavage. Control groups (control diet and HFD groups) were given an oral gavage of an equivalent volume of sterile MilliQ water. After 1 hour, mice were challenged with an oral glucose gavage (2 g/kg glucose dose), as previously described by Everard *et al*.^25^ Blood samples were collected at −30 min and 15 min to test total GLP-1 and insulin plasma concentration by using ELISA kits (Meso Scale Delivery, USA and Mercodia, Uppsala, Sweden, respectively). The insulin resistance index was determined by multiplying the area under the curve of both blood glucose and insulin in the plasma obtained by the oral glucose tolerance test (OGTT).

#### Chronic exenatide long-term treatment study in obese/diabetic mice

Eight-week-old male mice were randomly divided into seven groups (10 mice per group) and housed five per cage in a controlled environment (room temperature of 23°C ± 2°C, 12 h daylight cycle) with free access to sterile food (AIN93Mi; Research Diet) and sterile water. After a 2-week acclimation period, mice underwent 8 weeks of HFD or a normal control diet (Control). After this period, mice were treated daily at 16:00 pm (i) orally with exenatide in solution or encapsulated within RM-LNC (500 µg/kg dose) (EXE-RM-LNC) or the corresponding concentration of unloaded RM-LNC or (ii) subcutaneously with exenatide in solution (10 µg/kg) (EXE s.c.) or as Byetta (10 µg/kg). Control groups (healthy and HFD) were treated orally daily with an equivalent volume of sterile Milli-Q water. At the end of the treatment period, animals were anaesthetised with isoflurane (Forene, Abbott, England), and blood was sampled from both the portal and the cava veins. After exsanguination, mice were euthanised by cervical dislocation. Subcutaneous adipose tissue, liver and spleen were precisely dissected, weighed and immediately immersed in liquid nitrogen followed by storage at −80°C for further analysis or preserved in 4% paraformaldehyde for histological analysis (liver). Total lipids were measured after extraction with chloroform-methanol according to a modified Folch method,^26^ as previously described.^25^ Triglyceride and cholesterol concentrations were measured using a kit coupling an enzymatic reaction and spectrophotometric detection of the final product (Diasys Diagnostic and System, Holzheim, Germany). All samples were run in duplicate.

Additional protocols and procedures are described in online supplementary methods section.

### Statistical analysis

GraphPad Prism 7 programme (California, USA) was used to perform the statistical analyses. For all analyses and for each group, any exclusions were supported by the use of the Grubbs test for outlier detection. Values were normalised by log-transformation when variances were significantly different between groups before conducting the analysis. Two-way or one-way ANOVA followed by Tukey’s post hoc test was applied for comparisons among multiple groups. If the variance differed significantly between groups even after normalisation, a non-parametric test was performed. The results are expressed as the mean±standard error of the mean (SEM). A difference of p<0.05 was considered statistically significant.
