# Supplementary material for: Novel strategy for oral peptide delivery in incretin-based diabetes treatment
Source: Gut. 2019 Aug 10;69(5):911–9. doi: 10.1136/gutjnl-2019-319146 (PMC7229891; doi:10.1136/gutjnl-2019-319146)
Supplement: Supplementary data [file gutjnl-2019-319146supp001.pdf]

SUPPLEMENTARY FIGURES AND MATERIALS AND METHODS

Supplementary figures/tables

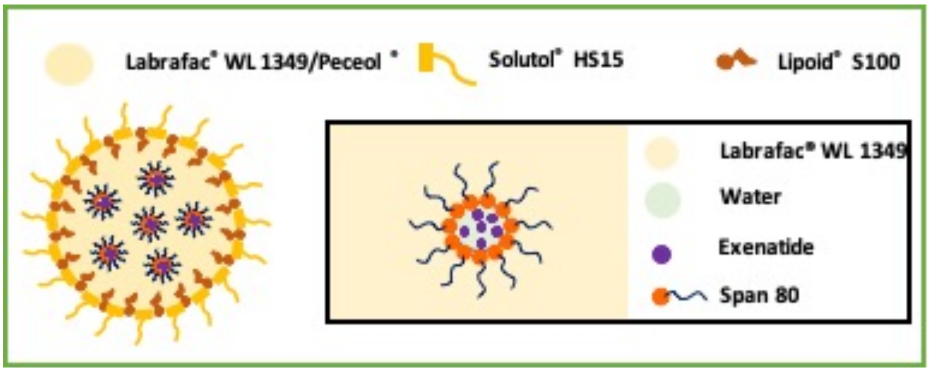

Supplementary Figure S1. A schematic representation of EXE RM LNC.

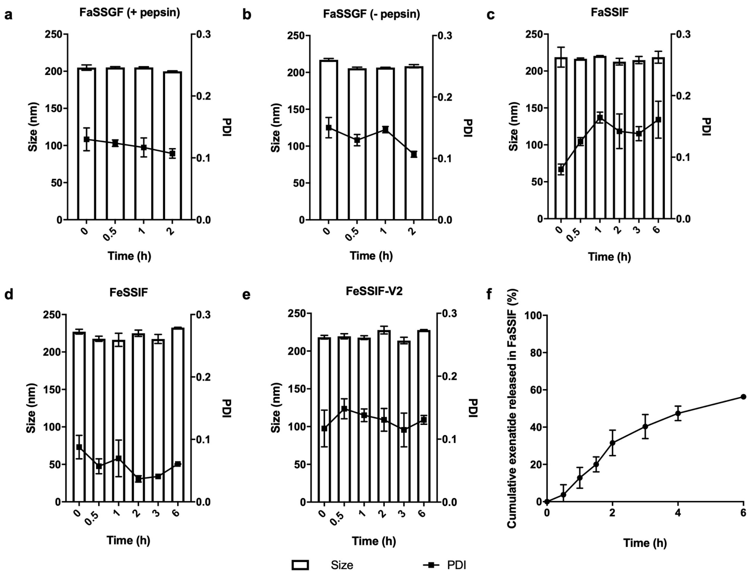

Supplementary Figure S2. Nanocapsule stability and exenatide release in biomimetic intestinal fluids. Particle size and PDI of EXE RM LNC variation following incubation in FaSSGF in the absence **a** and in the presence of pepsin **b**, in FaSSIF **c**, in FeSSIF **d** and in FeSSIF-V2 **e** at predetermined time intervals (mean ± SEM; n=9). The particle size of the EXE RM LNC was not significantly altered in any case (P>0.05) and exhibited monodispersity in the assayed media (PDI<0.2). **f** Cumulative exenatide release profile in FaSSIF (pH 6.5) at 37 °C over 6 h as measured by HPLC (mean ± SEM; n=9).

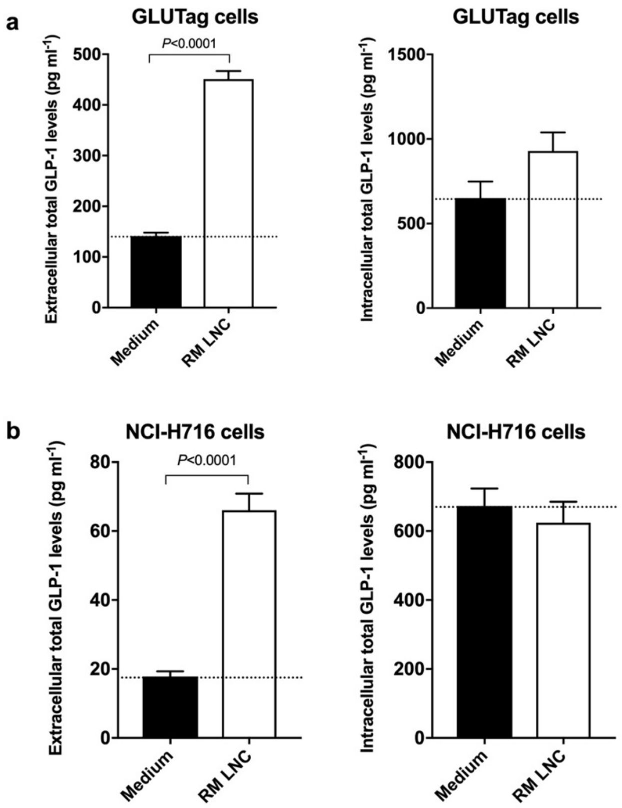

Supplementary Figure S3. GLP-1 secretion *in vitro* in GLUTag and NCl-H716 cells. **a** Extracellular (left) and intracellular (right) total GLP-1 levels measured by ELISA after 2 h of coincubation with RM LNC in GLUTag cells (murine L-cells). **b** Extracellular (left) and intracellular (right) total GLP-1 levels measured by ELISA after 2 h of coincubation with RM LNC in NCl-H716 cells (human L-cells). Data are presented as the mean ± SEM (n=12). *P* values were determined by Student's *t* test. The data correspond to three independent experiments.

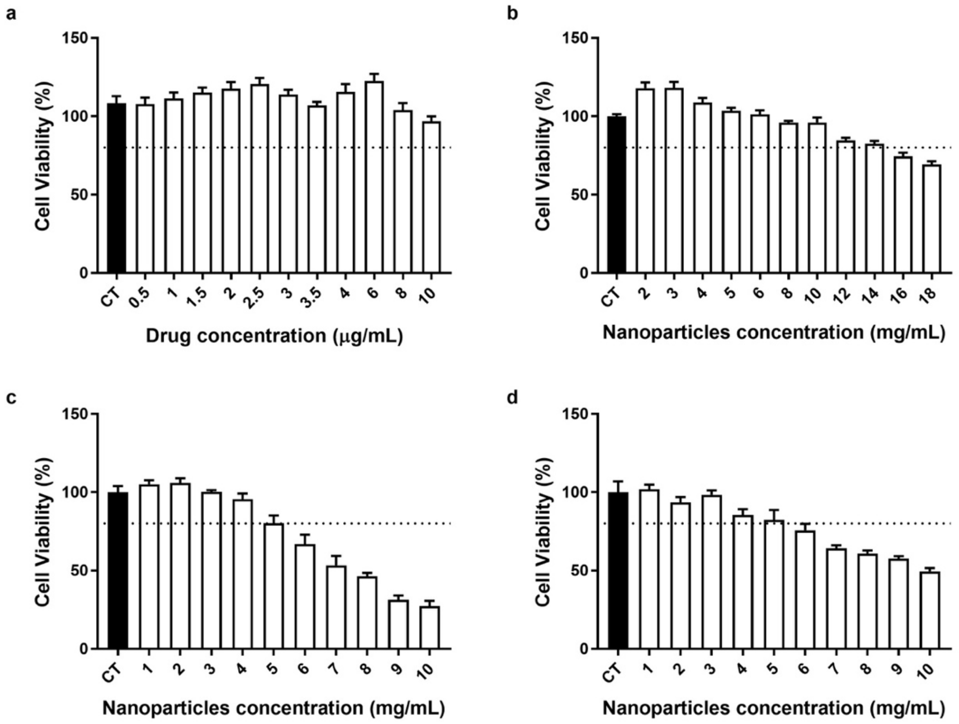

Supplementary Figure S4. *In vitro* cytotoxicity studies in L-cells and enterocyte-like cells. Cell viability of EXE RM LNC on Caco-2 cells after incubation for 2 h at 37°C expressed as cell viability with respect to drug concentration **a** and to nanoparticle concentration **b**. Cell viability of RM LNC on GLUTag cells **c** and human NCI-H716 cells **d** after coincubation with increasing nanocapsule concentrations (1 mg/mL to 10 mg/mL) for 2 h. Data are shown as the mean ± SEM (n=9). The dotted line corresponds to 80% availability. The data correspond to three independent experiments.

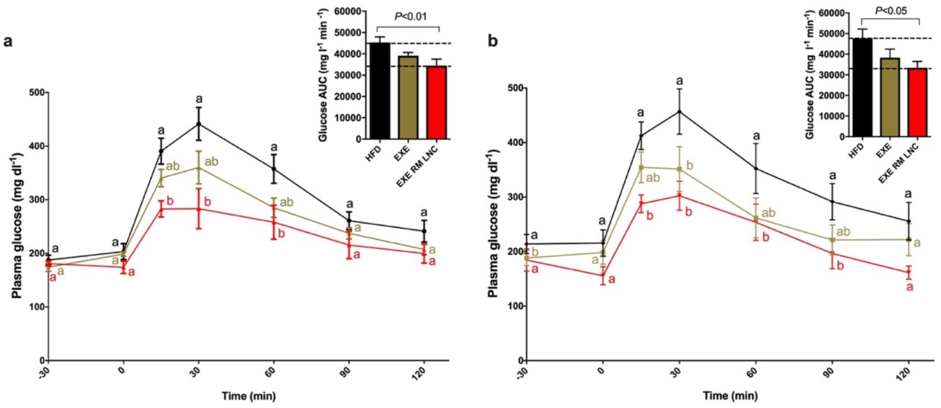

Supplementary Figure S5. OGTT evaluation of EXE RM LNC in 8- and 10-week HFD-induced diabetic mice. **a** Plasma glucose levels and AUC following a 2 g/kg glucose oral challenge measured in mice (C57BL6/J mice) fed a control diet and a HFD (8 weeks) and **b** fed a control diet and a HFD (10 weeks). Data are presented as the mean ± SEM (n= 7-8). Values with different superscript letters are significantly different *(P*<0.05). *P* values were determined by two-way ANOVA for the OGTT and by one-way ANOVA followed by Tukey's post hoc test for comparing the AUC between groups.

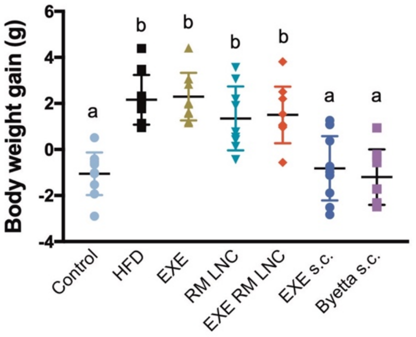

Supplementary Figure S6. Body weight gain following chronic/long-term administration of exenatide in obese and diabetic mice. Body weight gain (g). Data are presented as the mean ± SEM (n=10). *P* values were determined following by a one-way ANOVA followed by Tukey's post hoc test.

Supplementary Table S1. Composition of 220 nm EXE RM LNC.

Composition	Reverse micelles (RM)	Lipid nanocapsules (LNC)
**Drug solution (µL)**	**50**	**/**
**Span® 80 (mg)**	**100**	**/**
**Labrafac® WL 1349 (mg)**	**500**	**769.5**
**Peceol® (mg)**	**/**	**85.5**
**Lipoid® S100 (mg)**	**/**	**13.4**
**Solutol® HS15 (mg)**	**/**	**120**
**Sodium chloride (mg)**	**/**	**50**
**MilliQ water (µL)**	**/**	**1025**
**Cold MilliQ water (µL)**	**/**	**2500**

Supplementary Table S2. Physicochemical characterization of EXE-loaded or unloaded RM LNC (PDI: polydispersity index; EE: encapsulation efficiency).

Formulations	Mean Size (nm)	PDI	Zeta potential (mV)	EE (%)
EXE RM LNC	224.4 ± 3.2	0.185 ± 0.013	-3.06 ± 0.40	84.95±3.76
RM LNC	215.7 ± 3.3	0.165 ± 0.012	-2.993 ± 0.29	-

Supplementary Table S3. Composition of biomimetic intestinal fluids.

Composition	FaSSGF Concentration (mM)	FaSSIF Concentration (mM)	FeSSIF Concentration (mM)	FeSSIF-V2 Concentration (mM)
Sodium taurocholate	0.08	3	15	10
Lecithin	0.02	0.75	3.75	2
Glycerol monooleate	/	/	/	5
Sodium oleate	/	/	/	0.8
Sodium chloride	34.2	34.2	203.18	126
Sodium monobasic Phosphate anhydrous	/	28.65	/	/
Sodium hydroxide	/	8.71	101.02	82
Acetic acid	/	/	144.04	/
Maleic acid	/	/	/	55
Pepsin	0.1 mg/ml	/	/	/
pH	1.6	6.5	5	5.8

Supplementary Table S4. Pharmacokinetic parameters of the *in vivo* study in obese/diabetic mice for s.c. exenatide (50 μg/kg), oral exenatide (500 μg/kg) and oral EXE RM LNC (500 μg/kg). *P* values were significantly different *(****P*<0.01 and ***P*<0.001) according to a one-way ANOVA followed by Tukey's post hoc test. Significance is expressed compared to the exenatide oral group.

**Formulation**	**C_{max} (ng/mL)**	**T_{max} (h)**	**AUC_{0-8} (ng · h/mL)**	**$t_{1/2}$**	**FR (%)**
Exenatide s.c.	10.27 ± 6.82**	1.5	27.30 ± 12.46***	1.38 ± 0.36	100***
EXE LNC oral	8.73 ± 4.53**	0.5	11.79 ± 2.73***	1.68 ± 0.35	4.32 ± 0.95***
Exenatide oral	0.86 ± 0.93	0.5	3.71 ± 0.93	---	1.36 ± 0.34

Supplementary Table S5. Primer sequences for gene expression analyses by RT-qPCR

	Forward primer	Reverse primer
Rpl19	GAAGGTCAAAGGGAATGTGTTCA	CCTTGTCTGCCTTCAGCTTGT
F4/80	TGACAACCAGACGGCTTGTG	GCAGGCGAGGAAAAGATAGTGT
Cd11c	ACGTCAGTACAAGGAGATGTTGGA	ATCCTATTGCAGAATGCTTCTTTACC
Mcp1	GCAGTTAACGCCCCACTCA	TCCAGCCTACTCATTGGGATCA
Tnfa	TCGAGTGACAAGCCTGTAGCC	TTGAGATCCATGCCGTTGG
Fasn	CAGGCCCCTCTGTTAATTGG	TCCAGGGATAACAGCACCTT
Pparg	CTGCTCAAGTATGGTGTCCATGA	TGAGATGAGGACTCCATCTTTATTCA
Cpt1a	AGACCGTGAGGAACTCAAACCTAT	TGAAGAGTCGCTCCCACT

Rpl19, ribosomal protein L19; *F4/80* (also known as Adgre1), egf-like module-containing, mucin-like, hormone receptor-like1; *Cd11c* (also known as Itgax), clusters of differentiation; *Mcp1* (also known as Ccl2), monocyte chemotactic protein 1; *Tnfa*, tumor necrosis factor, alpha; *Fasn*, fatty acids synthase; *Pparg*, peroxisome proliferative activated receptor, gamma; *Cpt1a*, carnitine palmitoyltransferase 1, alpha.

Supplementary materials and methods

Materials

Labrafac® WL 1349 (caprylic/capric acid triglycerides) and Peceol® (oleic acid mono-, di- and triglycerides) were gifts from Gattefossé (Saint-Priest, France). Lipoid® S 100 (soybean lecithin at 94% of phosphatidylcholines) was a gift from Lipoid GmbH (Ludwigshafen, Germany). Solutol® HS15 (mixture of free PEG 660 and PEG 660 12-hydroxystearate, Mw 870 Da) and Span® 80 (sorbitan oleate) were purchased from Sigma-Aldrich (St. Louis, USA). Sodium chloride (NaCl), lecithin, sodium taurocholate, pepsin, 3-(4,5- dimethyl-thiazol-2-yl)-2,5-diphenyl tetrazolium bromide (MTT), dimethyl sulfoxide (DMSO) and Triton-X 100 were purchased from Sigma-Aldrich (St. Louis, USA). Exenatide was purchased from Bachem (Bubendorf, Switzerland). Total GLP-1 (ver.2) and active GLP-1 assay kits were purchased from Meso Scale Discovery (Maryland, USA). An Exendin-4 enzyme immunoassay kit was purchased from Phoenix Europe GmbH (Karlsruhe, Germany). An Ultrasensitive Mouse Insulin ELISA Kit was purchased from Mercodia AB (Uppsala, Sweden). Matrigel™ was obtained from BD Bioscience (Belgium). Dipeptidyl peptidase IV (DPP-IV) inhibitor was purchased from Millipore (St. Charles, USA). Dulbecco's modified Eagle's medium (DMEM)-GlutaMAX (5.5 Mm glucose), Roswell Park Memorial Institute (RPMI)-1640 medium, penicillin-streptomycin (P/S), fetal bovine serum (FBS), phosphate buffered saline (PBS), trypsin (0.25%) containing ethylenediaminetetraacetic acid (EDTA, 0.02%) were also used and purchased from Thermo Fisher Scientific (Invitrogen, Belgium). All chemical regents utilized in this study were of analytical grade.

Quantification of exenatide

The exenatide encapsulated within RM LNC was quantified by high-performance liquid chromatography (HPLC, Shimadzu, Japan) using a gradient method as previously described by Shrestha et al. [13] Briefly, a Kinetex® EVO C18 column (100 Å, 2.6 μm, 150 x 4.6 mm) (Phenomenex, USA), with a security guard column (Phenomenex, USA) was used at room temperature. The aqueous mobile phase comprised 0.05% (v/v) trifluoroacetic acid (TFA) in water, and the organic mobile phase consisted of 0.05% (v/v) in acetonitrile. A gradient system was developed with an initial ratio of 10:90 (v/v, aqueous:organic phase) at a flow rate of 1 mL/min, which was linearly changed to 90:10 (v/v) over 10

min and kept constant for the next minute. Then, the ratio was linearly changed to the initial composition in the next 1.5 min and was stabilized for the last minute. The injection volume used was 20 μL ,and the detection wavelength used was 220 nm. The retention time was 5.9 min, and the limit of detection and limit of quantification were 1.1 ± 0.4 μg/mL and 3.3 ± 1.1 μg/mL, respectively.

Characterization of EXE RM LNC

EXE RM LNC were characterized by measuring their particle size and polydispersity index (PDI) by dynamic light scattering (DLS) using a Zetasizer Nano ZS (Malvern Instruments Ltd., Worcestershire, UK). The zeta potential was determined by laser Doppler velocimetry (LDV) using a Zetasizer Nano ZS. For the measurement, 5 μL of nanocapsule suspension was dispersed in 995 μL of ultrapure water. All measurements were performed in triplicate.

EXE RM LNC were also characterized based on their drug encapsulation efficiency (EE, %). To calculate the total drug content, 50 μL of EXE RM LNC were dissolved in 950 μL of methanol followed by strong vortexing. Free and encapsulated exenatide were separated by ultrafiltration using Amicon® centrifuge filters (MWCO 30 kDa, 4000g, 4°C, 20 min) (Millipore). Filtrates were further diluted using a 1:2 dilution factor. The exenatide in the filtrate and the exenatide dissolved in methanol were quantified using the above-described HPLC method. The EE was calculated using the following equation:

EE (%) = (total amount of exenatide – free exenatide) / (total amount of exenatide) ×100

Nanocapsule stability and drug release in stimulated gastrointestinal fluids

The *in vitro* stability of EXE LNC was tested in five different biomimetic media: Fasted State-Simulated Gastric Fluid (FaSSGF) with and without pepsin, Fasted State-Simulated Intestinal Fluid (FaSSIF), Fed State-Simulated Intestinal Fluid (FeSSIF) and FeSSIF version 2 (FeSSIF-V2) (biorelevant.com, UK). A detailed description of the composition of the simulated fluids used is presented in the Supplementary Material in Table S1. The influence of gastric and intestinal conditions on nanocapsule stability was evaluated based on the nanocapsule size and the PDI. EXE RM LNC were incubated in FaSSGF with and without pepsin, FaSSIF, FeSSIF and FeSSIF-V2 at 37 °C (100 μL of nanocapsules in 10 mL of media) under gentle stirring. At predetermined time intervals (0, 0.5, 1 and 2 h for stimulated gastric media and 0, 0.5, 1, 3 and 6 h for stimulated intestinal media and FeSSIF), samples were withdrawn and then analyzed by DLS.

***In vitro* drug release studies**

The drug release from EXE RM LNC was evaluated in FaSSGF in the absence of pepsin and in FaSSIF media for 2 h and 6 h, respectively. Studies were performed using the dialysis method. Briefly, 1 mL of EXE RM LNC was placed in disposable dialysis membranes (MWCO 100 kDa) (Float-A-Lyzer®G2, Microfloat, Spectrum labs, USA) and introduced into 50 mL falcon tubes containing 35 mL of medium at 37 °C under magnetic stirring. At predetermined times, 50 µL of sample was withdrawn and dissolved in 950 µL of methanol. The concentration of exenatide was determined by the HPLC, as described above.

***In vitro* cell studies**

Cell cultures

The human NCI-H716 L cell line was obtained from the American Type Culture Collection (ATCC) (Manassas, VA) and was used from passages 15 to 20. The completed media was composed of Roswell Park Memorial Institute (RPMI) 1640 medium with 1% (v/v) penicillin−streptomycin (P/S) and 10% (v/v) fetal bovine serum (FBS). Cells were suspended growth in 75 cm^2 flasks (Corning, Lowell, MA, USA) in an atmosphere of 5% CO_2/95% air (v/v) at 37°C. Some fresh medium was added every other day. After replacement of the medium, cultured were centrifuged and subsequently resuspended at a proper density.

The intestinal murine L cell line GLUTag was kindly provided by Dr. Daniel Drucker (University of Toronto, Canada). GLUTag cells were used from passages 16 to 29. Cells were grown in DMEM GlutaMAX (5.5 mM glucose) supplemented with 10% (v/v) inactivated FBS and 1% (v/v) P/S (complete DMEM medium) at 37 °C with 5% CO_2 supply. Cells were subcultured using trypsin (0.25%) containing EDTA (0.02%) every 4-5 days.

Caco-2 cells (clone-1) were kindly provided by Dr. Maria Rescigno (University of Milano-Bicocca, Milano, Italy) and used from passages 25 to 30. The Caco-2 cell line was maintained in medium consisting of DMEM supplemented with 10% (v/v), HyClone™ FBS, 1% (v/v) L-Glutamine, 1% (v/v) nonessential amino acids and 1% (v/v) P/S at 37 °C in an atmosphere of 10% CO_2/95% air (v/v). The medium was replaced every other day.

Cytotoxicity studies

In vitro cytotoxicity studies of EXE RM LNC were performed in Caco-2 cells based on the drug and nanocapsule concentration calculated as previously described using 3-(4,5-dimethylthiazol-2-yl)-(2,5-diphenyltetrazolium bromide) (MTT) colorimetric assay [26]. The influence of unloaded RM LNC on cell viability was also tested in GLUTag and NCI-H716 cells. Caco-2 cells (5×10^4 cells per well) were seeded in 96-well tissue culture plates (Costar Corning CellBIND Surface, USA) and were allowed to adhere overnight. For cytotoxicity studies in GLUTag and NCI-H716, 5×10^4 cells per well were seeded on Matrigel™-coated (10 µL/mL of medium) 96-well plates. After washing the plates with prewarmed PBS buffer (x3), 100 µL of EXE RM LNC at increasing drug concentrations (0.5-10 mg/mL), corresponding to increasing nanocapsule concentrations (2-18 mg/mL), were dispersed in DMEM (without FBS) and coincubated with Caco-2 cells for 2 h at 37 °C. Increasing unloaded RM LNC concentrations (from 1 mg/mL to 10 mg/mL) were dispersed in DMEM GlutaMAX mor RPMI-1640 medium (without FBS) and coincubated with GLUTag or NCI-H716 cells, respectively, at 37 °C for 2 h. After incubation, the supernatants were replaced by 100 µL of 0.5 mg/mL MTT for 3 h. The purple formazan crystals were dissolved in 200 µL of DMSO for absorbance determination at 560 nm using a MultiSksan EX plate reader (Thermo Fisher Scientific, USA). Cells with Triton-X 100 (100% dead) and cells with culture medium (100% alive) were considered positive and negative controls, respectively. Tests were performed in triplicate.

Animal studies

Effects on body composition and adipose tissue were evaluated by the weights of subcutaneous (SAT), epididymal (EAT), visceral adipose tissues (VAT) and brown adipose tissues (BAT) (mg). Levels of glucose and gastrointestinal hormones involved in food intake and body weight, including total GLP-1 (ELISA kit, Meso Scale Delivery, Gaithersburg, USA) and insulin (Ultrasensitive insulin ELISA, Mercodia, Uppsala, Sweden), were measured in peripheral blood and portal vein blood. Liver steatosis was visualized by oil red O staining. Liver tissue was embedded in Tissue-Tek Optimal Cutting Temperature compound (Sakura Europe, Leiden, Netherlands) and flash-frozen in cold isopentane. Five-micrometer-thick tissue sections were stained with oil red O staining for lipid content analysis. Five high-magnification fields (20x) were analyzed per mouse. Quantification of the mean droplet area was

performed using ImageJ software (Version 2.0.0-rc-69/1.52i, National Institutes of Health, Bethesda, Maryland, USA). The general morphology of the liver was assessed by hematoxylin and eosin (H&E)-stained sections. For the real-time quantitative PCR (qRT-PCR) analysis, the total RNA was isolated from tissues using TriPure reagent (Roche). Complementary DNA was prepared by reverse transcription of 1 µg total RNA using a Reverse Transcription System kit (Promega, Madison, Wisconsin, USA). Real-time PCR was performed with a CFX96 real-time PCR system and CFX Manager 3.1 software (Bio-Rad, Hercules, California, USA) using GoTaq® qPCR Master Mix (Promega, Madison, USA) for detection according to the manufacturer's instructions. Ribosomal protein L19 (*Rpl19*) was chosen as the housekeeping gene. All samples were run in duplicate in two 96-well reaction plates, and data were analyzed according the 2−ΔΔCT method. The identity and purity of the amplified product were assessed by melting curve analysis at the end of the amplification. Primer sequences for the targeted mouse genes are presented online in supplementary table 5.

Total GLP-1 secretion in normoglycemic mice

Normoglycemic mice (C57BL/6J male mice, 20–25 g, 10 weeks; Janvier Laboratories, France) were randomly divided into two groups with 8 mice each. Animals were fasted overnight before the experiments with free access to water. Mice were treated with blank RM LNC corresponding to ~1.62 mg/g nanoparticle dose. Control mice were treated by oral gavage with an equivalent volume of MilliQ water. Blood samples were withdrawn from the tip of the tail vein at 60 and 180 min after oral administration. Samples were collected in the presence of DPP-IV inhibitor (20 µL per mL of blood) and maintained on ice. Immediately after the study, blood samples were centrifuged (3,000 rpm, 10 min at 4 °C), and the plasma was kept frozen at −80 °C until analysis. The total GLP-1 levels were quantified using a Total GLP-1 ELISA kit. (Meso Scale Delivery, USA). The total GLP-1 plasma values are expressed as the fold-change compared with the untreated control group.

Pharmacokinetics study in normoglycemic and obese/diabetic mice

Eight-week-old male mice were randomly divided into three groups (10 mice per time point) and housed in a controlled environment (room temperature of 23 ± 2 °C, 12 h daylight cycle) with free access to food and sterile water. After two weeks of acclimation, mice underwent 3 weeks of HFD (60% fat). Prior to the experiments, mice were fasted overnight with free access to sterile Milli-Q water. Exenatide

in solution and EXE-RM-LNC were orally administered at a 500 μg/kg dose. Exenatide was also subcutaneously administered at a 50 μg/kg dose. At different time points (0, 0.5, 1, 1.5, 2, 4, 6 and 8 h), blood samples were collected from the tip of the tail vein. Blood samples were then centrifuged (1,500 *g*, 10 min at 4 °C), and exenatide plasma concentration was quantified using an ELISA kit (EK-070-94, Phoenix Europe GmbH, Karlsruhe, Germany). The relative bioavailability (FR %) of exenatide was calculated using the following equation:

$$FR(\%) = \frac{\mathrm{AUC_{oral}} \times \mathrm{Dose_{s.c.}}}{\mathrm{AUC_{s.c.}} \times \mathrm{Dose_{oral}}} \times 100$$

The pharmacokinetic parameters were analyzed using PKSolver.[27] In the case of normoglycemic mice, 4 mice per time point were used instead.
